# LightMG-Net: an efficient lightweight deep neural network for multiclass grading of retinal detachment using handcrafted statistical mechanisms

**DOI:** 10.1038/s41598-025-26152-4

**Published:** 2025-11-26

**Authors:** Sonal Yadav, R. Murugan, Balachandra Pattanaik, Tanveer Ahmed

**Affiliations:** 1 Bio-Medical Imaging Laboratory (BIOMIL), Department of Electronics and Communication Engineering, National Institute of Technology Silchar, Silchar, Assam 788010 India; 2 School of Electrical and Computer Engineering, Wollaga University, P.O. BOX 395, Nekemte, Ethiopia; 3 Saveetha School of Engineering, Saveetha Institute of Medical and Technical Sciences, Chennai, 602105 India; 4 Department of Ophthalmology, Silchar Medical College and Hospital, Silchar, Assam 788014 India

**Keywords:** Electrical and electronic engineering, Eye diseases

## Abstract

Retinal detachment is a severely curable eye condition that becomes a genuine factor for the increased visual acuity worldwide. If neglected, it may result significant visual impairment in individuals aged 60 to 69 years. The successful cure percentage of retinal detachment critically relies on early-stage diagnosis. If addressed early, almost 90% of people with retinal detachment can recover from vision loss. Consequently, it is imperative to classify retinal detachment patients in an early phase. We developed a novel optimized lightweight multiclass retinal detachment grading model named LightMG-Net, which utilizes the image and feature-oriented handcrafted techniques for best feature analysis and the Grey Wolf Optimization technique for automatic hyperparameter tuning of a lightweight convolutional neural network for multiclass grading of retinal detachment from fundus images. The proposed LightMG-Net model is applied on four commonly utilized online databases, such as the Retinal Image Bank, Cataract Image Dataset, Kaggle, and Eye Disease Retinal Image for validation. The proposed LightMG-Net model achieved the best classification accuracy, sensitivity, specificity, and area under the curve at 95.42%, 95.10%, 98.90%, and 0.9947, respectively. Experimental outcomes demonstrate the superiority of the proposed approach over existing baseline methodologies.

## Introduction

**Fig. 1:**
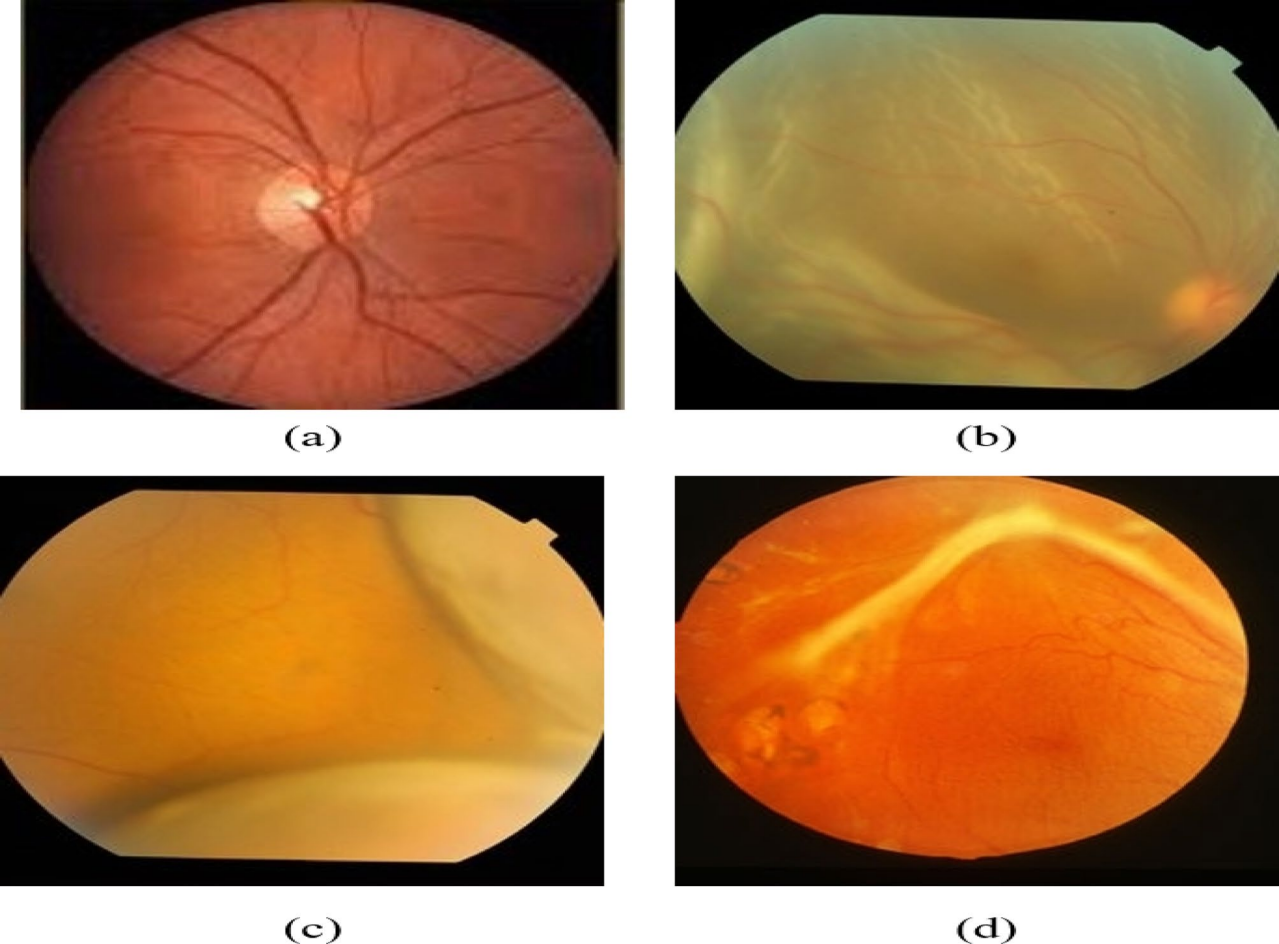
Sample images of (**a**) Healthy^10^, (**b**) Rhegmatogenous, (**c**) Exudative, and (**d**) Tractional RD fundus images^11^.

Retinal Detachment (RD) is a visual condition characterized by separating the neurosensory retina from its underlying vascular layer, which supplies oxygen and nutrients^1^. It is a serious vision-threatening condition; if not addressed promptly, it may result in irreversible visual loss. RD typically occurs in approximately 1 individual per 10,000 individuals aged 60 to 70 years^2,3^. The three main types of retinal detachment (RD) are rhegmatogenous retinal detachments (RRD), tractional retinal detachments (TRD), and exudative retinal detachments (ERD)^4^. RRD is the predominant type of RD, resulting from full-thickness ruptures in the retina induced by vitreoretinal traction^5^. The occurrence of RRD is roughly 17 patients per 10,000 individuals over the age range of 60^6^. TRD primarily occurs as a consequence of diabetes, which compromises the blood vessels in the supportive layer, resulting in the production of scar tissue^7^. In an ERD, the retina exhibits no tears or holes on its surface; however, fluid accumulates behind it, displacing it from the underlying supportive layer^8^. Overall, fluid accumulation in the sub-retinal space beneath the neurosensory retina is a distinguishing feature of all forms of RD^9^. Figure 1 shows a few samples of various types of RD.

RD often causes visual loss and sharply drops in visual acuity. Still, early screening of RD can raise the possibilities of visible gain and reattachment, mainly when the macula is not involved in detachment^12^. However, detecting RD is very difficult because it starts asymptomatically and rises gradually at the retinal periphery. The initial indications of RD are floaters, flashes of light, curtain-like shades, and peripheral vision become dark^13^. 17% of the patients attribute these symptoms to a problem with their contact lenses, glasses, or aging^14^. For that reason, patients seldom visit the ophthalmologists for a regular checkup promptly until the visual insight sharply drops^15^. An experienced ophthalmologist can detect the RD by examining the fundus image with mydriasis, which is tedious and labor-intensive^16^. These challenges are hurdles in implementing RD screening in underdeveloped areas with fewer experienced ophthalmologists.

Recently, Deep learning (DL) has played a significant role in ophthalmology in areas like glaucoma, diabetic retinopathy, and age-related macular degeneration^17^. Especially the recent advancement of Convolutional Neural Networks (CNNs). CNNs have shown a powerful capacity to extract and combine spatial features, resulting in high-level features that are more representative than manually constructed features^18^. These CNN-based automated RD detection screenings can be implemented efficiently over a large population in a reduced timeframe.

Therefore, in this work, we developed an automatic lightweight CNN-based detection model capable of recognizing RD instantly and accurately.

The novelty of the proposed LightMG-Network is developing a lightweight convolutional neural network with clinically standard statistical handcrafted features and Grey Wolf Optimization (GWO) for hyperparameter tuning for multiclass RD grading. The state-of-the-art methods for RD screening mainly deal with feature extraction using deep neural networks without any handcrafted statistical features. The deep neural network benefits from a tremendous amount of training data; however, with ground-truthed datasets of limited size, it may fail to extract meaningful features on its own, which are essential for achieving better classification performance. We proposed a new lightweight convolutional neural network for feature extraction with a clinically standard statistical handcrafted mechanism to alleviate such a problem.

## Major contributions

The key contributions of this work are listed below:

- This work proposes an automated optimized lightweight convolutional neural network to identify the severity of retinal detachment disease.
- This work utilizes image and feature-oriented handcrafted statistical mechanisms to obtain rich features for better image analysis.
- The hyperparameters of the lightweight convolutional neural network are tuned by Grey Wolf Optimization to achieve the most accurate results for the multiclass grading of RD disease.
- This work is implemented on four publicly accessible databases to validate the performance of the proposed model, and the model’s efficacy and generalization were assessed using ablation experiments.

The manuscript arrangement is as follows: The second section pertains to related work, which will mostly expound upon the most recent scientific advancements and provide comprehensive details of each work. The third section comprehensively delineates the image and feature-level handcrafted techniques, proposed lightMG-Net model architecture, and a detailed description of the GWO technique. The fourth part comprises the experimental section, which encompasses an overview of the experimental environment, comparative analysis, and an ablation examination. The conclusion and future direction are provided in the last part of the manuscript.

## Related works

A thorough review of some of the DL-related works published in the last 5 years for RD classification is presented in this section.

Ohsugi et al.^19^ proposed a multilayer CNN model for early diagnosis of RRD through ultra-wide field (UWF) color fundus images. This framework demonstrated better specificity and sensitivity values of 89.39% and 97.5% respectively. According to these results, this work compared the support vector machine (SVM) model with the deep learning model and noticed that DL outperformed the SVM model in sensitivity and specificity values. Masumoto et al.^20^ trained an ensemble model for the screening of RRD. They trained nine different neural networks (DenseNet201, DenseNet121, ResNet50, VGG16, InceptionV3, VGG19, Xception, InceptionResNetV2, and DenseNet169) using 693 bullous RRD, 125 non-bullous RRD and 600 healthy images. Zhongwen et al.^21^ developed a deep-learning framework for detecting retinal fractures and lattice degeneration on UWF color fundus images. This model utilized a training and validation dataset including 5606 UWF images from 2566 individuals. This work employed a test dataset including 750 images to assess the efficacy of the twelve pre-trained models. The models were developed utilizing four distinct pre-trained algorithms: InceptionResNetV2, VGG16, InceptionV3, and ResNet50. Moreover, this work utilized three preprocessing techniques namely original, histogram-equalized, and augmented images. Li et al.^22^ proposed a hybridized pre-trained method for the prediction of the macula on/off RD and RD via UWF fundus images. The training samples utilized by the first framework are around 11,087 UWF fundus images, while the second method utilized 1771 ultra-wide fundus images. The sensitivity and sensitivity of both networks closely matched the performance of ophthalmologists having expertise of 3 to 5 years. Parra et al.^23^ suggested DL-based algorithms for the identification of peripheral retinal fractures using Optomap UWF fundus images. In this work, the AlexNet and SqueezeNet DL models were trained using 3 distinct mini batch sizes of 8, 16, and 32 for the identification of retinal cracks. Zhang et al.^24^ suggested a convolutional neural network termed seResNext50. The main objective of this model is to detect retinal fractures, RD, and lattice degeneration on tessellated eyes utilizing UWF fundus images. The seResNext50 model was trained on the original alongside two preprocessing techniques images such as cropping and rescaling images. A distinct dataset of 189 fundus images was employed to evaluate the model’s performance in comparison to previous models. Zhouetal.^25^ introduced two pre-trained Inceptionresnet50 and Resnet50 networks to identify recurrent retinal detachment via UWF fundus images following retinal reattachment therapy. Furthermore, the efficacy of this work was evaluated and compared against human ophthalmologists utilizing performance metrics of area under the curve (AUC), sensitivity, and accuracy using a prospective sample. Fung et al.^26^ utilized a DL architecture known as Inceptionv3 to determine the anatomic results of RRD surgery. The achieved sensitivity, specificity, and AUC of this framework were, 73.3%, 96.0%, and 0.94, respectively. Tang et al.^27^ devised a multi-label-based approach to automatically identify the RRD and its related diseases (lattice degeneration, central retinal tears, and retinal fractures) on UWF color fundus images. This study utilized InceptionResNet-V2, Inception-V3, and Xception as base networks, and implemented an ensemble model by unweighted averaging to improve performance by amalgamating the results of the base models. To address the class imbalance, cost-sensitive learning with a weighted binary cross-entropy loss function was employed to eliminate this issue. The t-distributed stochastic n-Neighbor Embedding was utilized to demonstrate the distinction of the neural networks. Gradient-weighted class activation mapping and guided backpropagation heat mapping were employed to clarify the predictions produced by the neural networks. Anitha et al.^28^ introduced a hybrid ML strategy employing the Levy flight-based atom search optimization (LFASO) method. The image pre-processing workflow includes scaling, image normalization, masking, data augmentation (DA), and segmentation. All retinal images are standardized by resizing to preserve consistent proportions, and this normalization improves performance and convergence. The DA technique enhances variety, while segmentation focuses on the area of interest. Subsequently, the meaningful properties are extracted from the pre-processed retinal images using a ResNet18 DL network. The LFASO approach is employed for the identification of the most salient deep features for RD classification. Hybrid machine learning approaches, including Gradient Boosting Machine, Random Forest, and SVM, are utilized to attain enhanced accuracy. Ayesha et al.^29^ presented novel federated CNN (FedCNN) algorithms for the automated detection of RD-based strabismus disease through eye-tracking datasets to improve diagnostic precision in ophthalmology. The FedCNN model integrates CNN with eXtreme Gradient Boosting (XGBoost) to extract the relevant features from the raw eye data and employs gaze deviation images to monitor dynamic eye movements.

A thorough examination of existing approaches reveals significant research gaps that must be addressed. The identified research gaps are: (1) The previous approaches exhibit computational complexity and require more computational resources. (2) Low-contrast RD lesions, alterations in lesion morphology, and irregularities can diminish the efficacy of precise feature extraction. (3) No studies have utilized the deep learning model for multiclass RD diagnosis. This work introduces a novel multiclass lightweight CNN framework that effectively addresses all research gaps while achieving superior classification outcomes.

## Methods

**Fig. 2:**
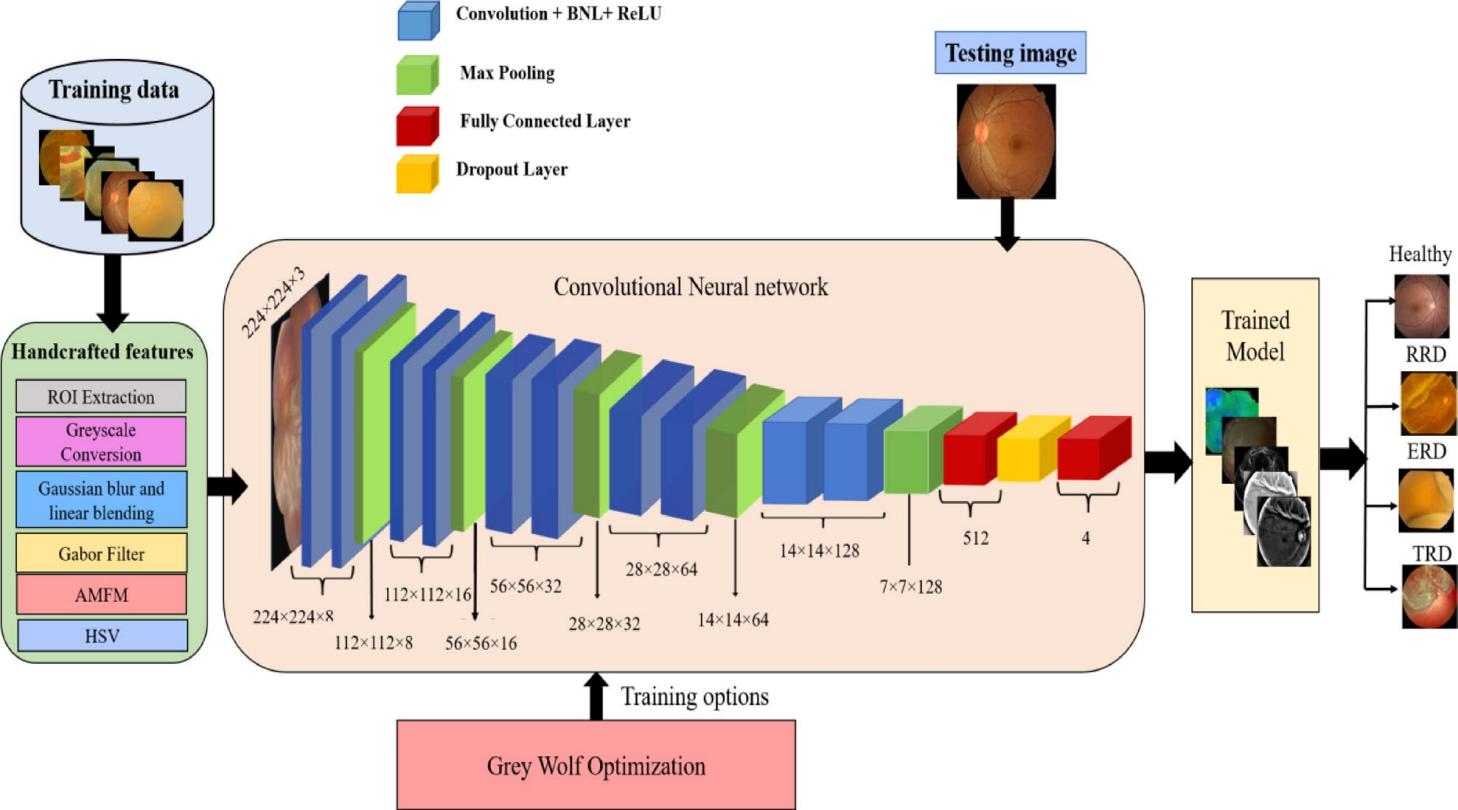
The flow diagram of the proposed LightMG-Net model.

In this work, we have developed a DL model called LightMG-Net for multiclass grading of RD disease. This section comprises four main stages: Database annotation, Image, and feature-oriented handcrafted approaches, a detailed description of the proposed LightMG-Net model, hyperparameter tuning using GWO, and multiclass classification based on optimal solutions provided by the GWO algorithm. This work employed image and feature-based handcrafted methods alongside a GWO algorithm to extract optimal hyperparameters, enhancing the proposed model’s performance. Figure 2 depicts the detailed structure of the proposed model.

### Database annotation

Due to the shortage of multiclass RD-labeled images on publicly available databases. It necessitates us to depend on multiple database sources for the collection of different types of RD fundus images. In the proposed work, we employed four online datasets: Retinal Image Bank (RIB)^11^, Cataract Image Dataset (CID)^10^, Kaggle^30^, and Eye Diseases Retinal Images (EDRI)^31^. The detailed description of these repositories are given in Table 1.

**Table 1:** Database utilized for RD multiclass grading.

Database	Total images	Resolution	Classes	ERD	Healthy	RRD	TRD	Annotation quality
RIB	30,370	643 × 427	4	98	–	170	65	Expert-graded fundus images
CID	601	300 × 200	4	–	300	–	–	Clinician-labeled healthy images
Kaggle	1000	3046 × 2572	39	–	38	57	–	Expert-graded fundus images
EDRI	1000	512 × 512	4	–	262	–	–	Labeled by ophthalmologists

#### Retinal image bank

The Retinal Image Bank is an initiative by the American Society of Retina Specialists that was launched online in August 2012. This platform acquires diverse fundus images of retinal diseases. This platform provides a repository of over 30,370 fundus images illustrating various types of retinal disorders. This work utilized 333 different forms of RD disease fundus images in JPG format to implement the proposed lightweight model. The images were captured utilizing fundus cameras featuring a 30-degree field of view. The images display a 643×427 resolution and are captured by an expert ophthalmologist.

#### Cataract image dataset

The Cataract Image Dataset also referred to as the Cataract and normal eye image dataset for cataract identification, was publicly provided in 2021 by JR2NGB.This database is accessible on GitHub under the name retinal_dataset. This dataset has four unique categories: (1) normal, (2) cataract, (3) glaucoma, and (4) retinal illness. This database comprises a total of 601 fundus images across all categories. We have obtained merely 300 standard fundus images from this collection. The images possess dimensions of 300×200 and are in PNG format. Furthermore, the medical information of the patients is included for each image in this repository.

#### Kaggle

The Kaggle database is referred to as 1000 Fundus Images encompassing 39 ocular disease types. This collection comprises 1000 fundus images representing 39 categories of various ocular diseases, sourced from the Joint Shantou International Eye Centre in Shantou City, Guangdong Province, China. This database was uploaded by Linchundan in 2018.We employed 95 fundus photos (38 from the healthy class and 57 from the RRD class) to develop the proposed lightweight model. The dimensions of these fundus photos are 3046×2572 pixels and they are in JPG format.

#### Eye diseases retinal images

The Eye Diseases Retinal Images were published on the Kaggle platform in 2022 by GUNA VENKAT DODDI. The collection consists of retinal images categorized into four types: Normal, Cataract, Glaucoma, and Diabetic Retinopathy. Each category has approximately 1000 retinal fundus photos. The present dataset has been modified from the original dataset to visualize the retinal defects more clearly. We have obtained 262 high-resolution normal fundus images, each with dimensions of 512×512 in JPG format. A fundus image of each eye was obtained for every patient across all categories in this database.

Although the images collected from the above repositories possess varying dimensions. Consequently, all images were resized to 224 ×224 ×3 pixels for the sake of statistical analysis.

### Handcrafted statistical mechanism

Handcrafted statistical approaches are essential for enhancing the quality and precision of fundus picture analysis. These techniques are crucial for improving image features and diminishing noise, which can obscure significant details. Some noise reduction techniques based on filtering methods are median or Wiener filters which aid in removing artifacts that may adversely affect the subsequent analysis. Transforming RGB images into grayscale is a prevalent procedure to diminish computing complexity while preserving critical information. Alternative techniques, like contrast enhancement and histogram equalization, enrich the visibility of features such as infected lesions and tears for classification tasks. This work utilized handcrafted techniques at both the image and feature levels, including the extraction of regions of interest (ROI), conversion of fundus images to grayscale, then linear blending with Gaussian blurred images, amplitude-frequency modulation (AM-FM) techniques, Gabor filters, and conversion from RGB to HSV.

### Image-level handcrafted approaches

**Fig. 3:**
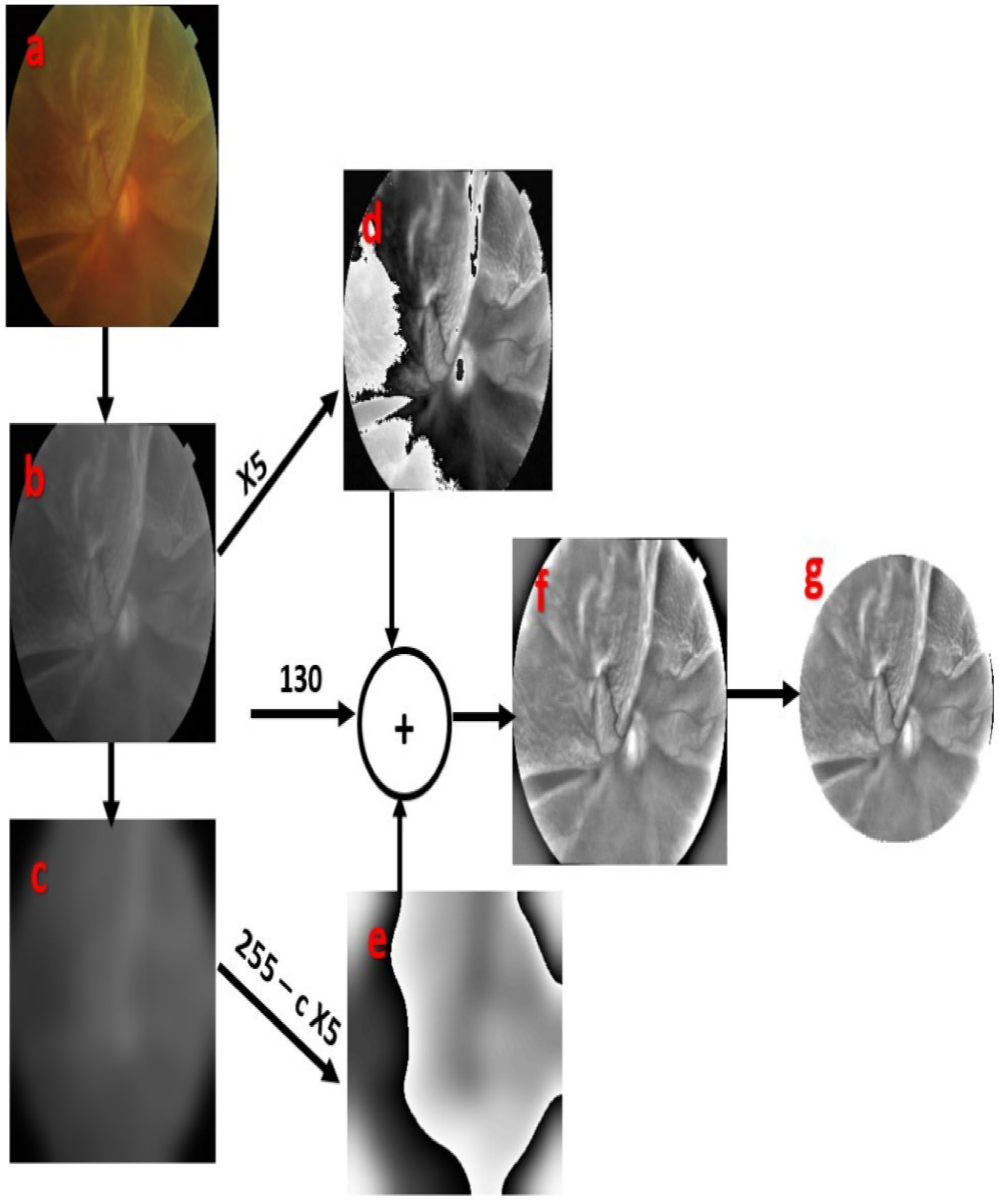
Image-oriented handcrafted steps applied on the raw fundus images.

The entire dataset has been converted to grayscale images to bring all the images to the same color palette. Additionally, several other image pre-processing approaches like Gaussian blur and linear blending were employed so that the deformities pertaining to RD become more prominent. One of the main advantages of converting the entire dataset to grayscale images is reduced complexity. Since, grayscale images only have a single channel, the mathematical computations have been reduced significantly in contrast to the original RGB images, thereby leading to faster gradient descent and significantly reduced training time. Figure 3a is a randomly chosen fundus image from the dataset. It is first converted into a grayscale image, as shown in Fig. 3b. The formula used to convert an RGB image to grayscale is given in equation (1):

1 $$Y = 0.{299}R + 0.{587}G + 0.{114}B$$

where R, G, and B are the RGB values corresponding to each pixel, and Y is the resulting pixel of the grayscale image. Then, a blurred image, i.e., Fig. 3c, is produced by applying a Gaussian Blur to the grayscale image. When a blur is applied, the color gradient from one pixel to another pixel is reduced, thereby creating the “smoothing” effect. A blur is essentially a low-pass filter that removes noise that exists in the RD fundus images. In Gaussian blur, a group of pixels surrounding the pixel to be filtered is considered. Then, we consider a kernel/convolution matrix of the same size as the rectangular group of pixels. The values of the kernel are based on the 2D Gaussian function, which is given by the equation (2):

2 $${(}G\left( {x,y} \right) = \frac{1}{{2\pi \sigma^{2} }}e^{{ - \left( {\frac{{x^{2} + \mu^{2} }}{{2\sigma^{2} }}} \right)}} .$$

From the above equation, we can see that the value will be maximum at its center, i.e., at x = 0 and y = 0. As we go further away from the center, the kernel values decrease. So, we can infer that the center pixel will be given the highest weightage, and as we go further away, the weightage decreases. This kernel will be used to perform a convolution with the rectangular group of pixels, and the resulting value will replace the center pixel of the rectangle. We used a kernel of size 99 × 99 and a standard deviation of 10.

Figure 3d is formed by multiplying a scalar value of 5 by each pixel of the grayscale image. The resultant image is essentially a brighter version of the grayscale image. It is to be noted that whenever a pixel value goes beyond 255, it is capping it to 255. Figure 3e is the negative transformation of the blurred image. In negative transformation, the bright pixels are converted to dark pixels and vice-versa. Firstly, a scalar 5 is multiplied by each pixel of Fig. 3c. Then, each pixel of the resulting image is subtracted from 255, and the negative image is reconstructed. Now, both Fig. 3d, e are linearly blended, which is simply the addition of each pixel of Fig. 3d with the corresponding pixel of Fig. 3e at a particular location. Then, an offset of 130° is added to the resultant image to brighten the image. Finally, Fig. 3f is circularly cropped to remove the background. In this way, we extract the relevant ROI of RD by removing the irrelevant background, as shown in Fig. 3g.

### Feature-level handcrafted approaches

**Fig. 4:**
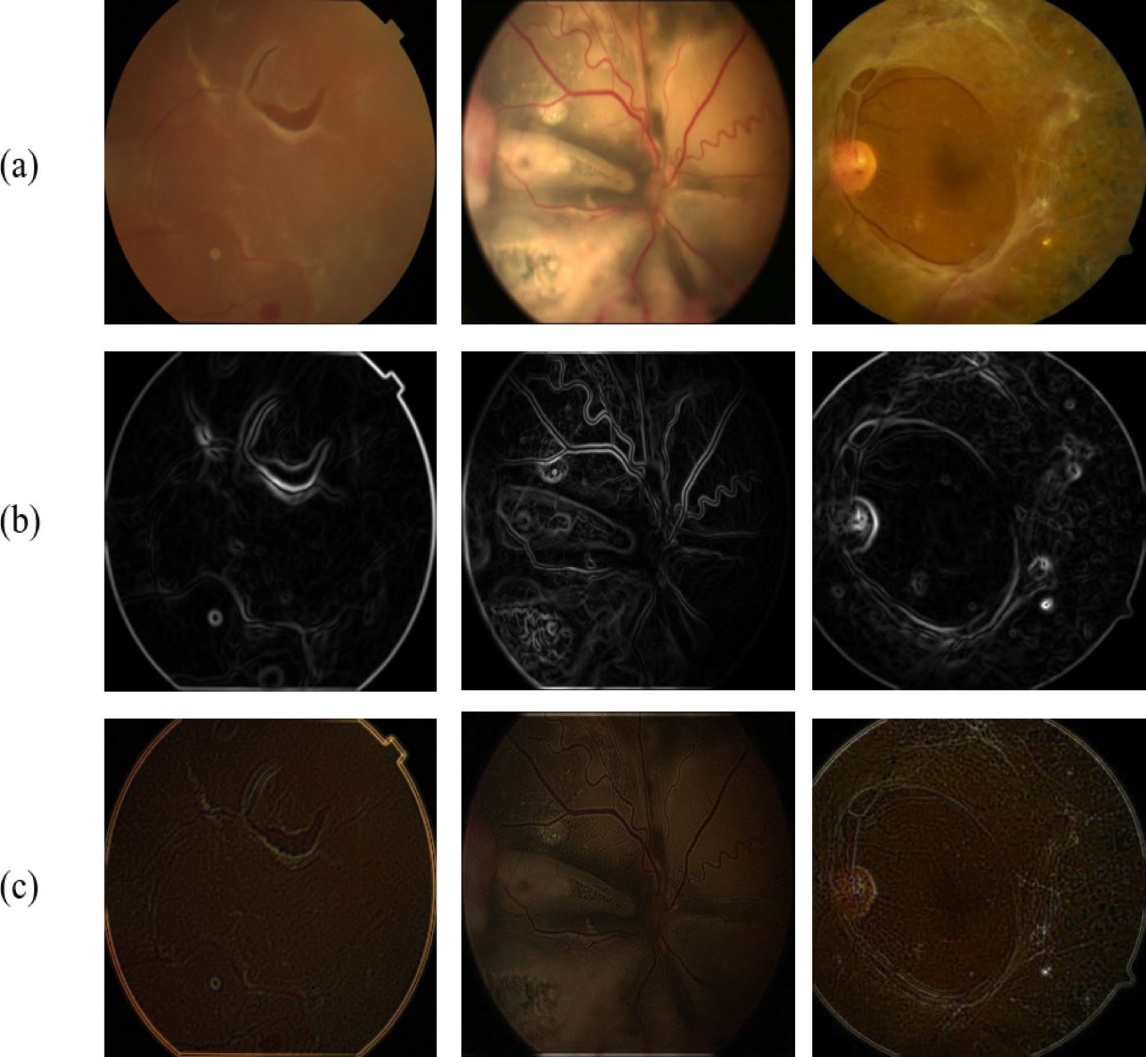
(**a**) The original fundus images, while (**b**), and (**c**) illustrate AM-FM and Gabor filter decomposed fundus images, respectively.

The amplitude-frequency modulation (AM-FM) technique retrieves information from different forms of RD fundus images as shown in Fig. 4a by decomposing the green channel at various scales into AM-FM components that denote the intensity and spatial variation of the fundus image, respectively^32^. The image is decomposed into AM-FM components using a series of Gaussian filters at varying scales with distinct standard deviations and sigma values as shown in Eqs. (3) and (4). These filters refine the image at various intensities, enabling variation to be captured a cross-multiple scales. For amplitude extraction, the amplitude component of the image at each scale is obtained by computing the absolute value of the smoothed image. Meanwhile, the frequency component is determined by computing the gradient of the smoothed image. After extracting amplitude and frequency features at each scale, we combined them to represent the texture and intensity of the retinal structures present in the image^33^ as shown in Fig. 4b.

Consider I(x,y) is the input green channel of the RD fundus image. At every scales, a Gaussian filter G_s_(x,y) with standard deviation *σ*_*s*_ is employed:

3 $$I_{s} \left( {x,y} \right) = I\left( {x,y} \right) * G_{s} (x,y)$$

where * represents convolution.

4 $$G_{s} \left( {x,y} \right) = \frac{1}{{2\pi \sigma_{s}^{2} }}{\text{exp}}\left( { - \frac{{x^{2} + y^{2} }}{{2\sigma_{s}^{2} }}} \right)$$

The amplitude component A_s_(x,y) of RD fundus images at s scale is achieved by the absolute value of the smoothed image as shown in Eq. (5).

5 $${\text{A}}_{{\text{s}}} \left( {{\text{x}},{\text{y}}} \right) = \left| {{\text{I}}_{{\text{s}}} \left( {{\text{x}},{\text{y}}} \right)} \right|$$

The frequency components F_s_(x,y) of RD fundus images is calculated by the gradient of the smoothed images as depicted in equation (6).

6 $$\vec{f}_{s} \left( {x,y} \right) = \nabla I_{s} \left( {x,y} \right) = \left( {\frac{{\partial I_{s} }}{\partial x},\frac{{\partial I_{s} }}{\partial y}} \right)$$

7 $$F_{s} \left( {x,y} \right) = \left\| {\nabla I_{s} \left( {x,y} \right)} \right\| = \sqrt {\left( {\frac{{\partial I_{s} }}{\partial x}} \right)^{2} + \left( {\frac{{\partial I_{s} }}{\partial y}} \right)^{2} }$$

Upon calculating A_s_(x,y) and F_s_(x,y) over several scales s 1,2,…,S, the texture and intensity characteristics are amalgamated commonly through stacking or concatenation of the features as shown in Eq. (8).

8 $${\text{Feature}}\;{\text{Vector}}\left( {x,y} \right) = \left[ {A_{{1}} \left( {x,y} \right),F_{{1}} \left( {x,y} \right), A_{{2}} \left( {x,y} \right),F_{{2}} (x,y), \ldots ,A_{S} \left( {x,y} \right),F_{S} (x,y)} \right]$$

The second approach employed is Gabor filters^34^, which are linear filters composed of sine and cosine functions modulated using a Gaussian kernel. The Gabor filter banks have utilized the spectral method to extract texture characteristics from RD color fundus images. The Gabor filter bank comprises four frequency values, six orientations, and two scales. The textures of RD fundus images are decomposed into characteristics via the Gabor filter bank as shown in equations (9)-(11) and Fig. 4c. The Gabor filter bank retrieved 48 characteristics from each image.

9 $$G\left( {i,j} \right) = \frac{1}{{2\pi \sigma_{i} \sigma_{j} }} \times e^{{ - \left( {\frac{{i^{{t^{2} }} }}{{2\sigma_{i}^{2} }} + \frac{{j^{{t^{2} }} }}{{2\sigma_{j}^{2} }}} \right)}}$$

10 $$i^{\prime } = a^{ - m} \left( {{\text{icos}}\left( {\theta_{n} } \right) + {\text{jsin}}\left( {\theta_{n} } \right)} \right)$$

11 $$j^{\prime } = a^{ - m} \left( { - i{\text{sin}}\left( {\theta_{n} } \right) + j{\text{cos}}\left( {\theta_{n} } \right)} \right)$$

In this context, σ_i_ and σ_j_ denote the window function dimensions, whereas W specifies the function’s frequency. a > 1 represents the scaling factor, with a predetermined value of 2 selected for this work. $\theta_{n}$ = $\frac{n\pi }{M}$, m = 0, 1,…,(S − 1), and n ranges from 0 to M—1. M and S denote the orientation and scale within the Gabor filter bank.

The third approach is the conversion of RGB images into the HSV color space. The hue saturation value (HSV) is a cylindrical color model that transforms the RGB color space into a more easily comprehensible format for humans. The Hue component denotes color information and offers more precise properties of color images for image classification. The saturation component denotes the perceived intensity of a specific color and quantifies the extent to which the actual color is blended with white. The value component indicates the perceived brightness of a particular color. The HSV color model is superior to RGB color images in terms of color fluctuations.

We leverage both spatial which includes grayscale conversion, ROI extraction, Gaussian blurring, linear blending, and Gabor filter) and frequency-based approaches (AM-FM) to extract the more informative features regarding texture and edges from the RD fundus images. After these handcrafted methods, we acquired 4475 fundus images. These disease-level feature images serve as input to an automated, tuned LightMG-Net model using GWO for multiclass severity classification of RD disease.

### Multiclass light-weight CNN model

We employed a multiclass RD grading tool utilizing a lightweight CNN architecture, including five convolutional blocks, two fully connected layers (FCL), and one dropout layer to diminish computational complexity while enhancing classification accuracy. The input layer of the multiclass lightweight CNN accepts images of dimensions 224×224×3 for effective training and learning with limited date. Each convolutional block comprises a convolution layer (Conv), ReLU activation, subsequent batch normalization layer (BNL) and max pooling layer (MPL).

The Conv layer of the proposed lightweight model is used to extract features from handcrafted images through multiple convolutional filters (CFs). Each CFs executes the convolution operation at each offset of the handcrafted images. The Conv layer comprises weights that require optimization through gradient descent training, which modifies the Conv layer parameters. The image characteristics derived from the Conv layer are represented in feature space with an on linear ReLU activation function. A BNL is employed with in the Conv layer and ReLU for normalizing the gradients and activations throughout the model. The MPL is utilized for down-sampling feature maps while preserving the most pertinent information. The MPL of the proposed lightweight model contains neither weights nor biases for training. The FCL is a classifier layer that classifies the characteristics retrieved from the Conv Layer and MPL into specific classes. Furthermore, after the five convolutional blocks, the first FCL has 512 neurons, accompanied by a dropout layer with a 30% dropout rate to mitigate overfitting. The second FCL comprises four neurons corresponding to the four disease classes: healthy, exudative, rhegmatogenous, and tractional RD. The final output layer classifies the input image into one of four classes via the softmax activation function.

Table 2 illustrates the layered design of the proposed LightMG-Net model. The proposed LightMG-Net model is lightweight because it uses fewer layers with fewer parameters and reduces computational complexity by employing a 3×3 filter size instead of using (5×5 or 7×7). Our proposed LightMG-Net model utilized a progressive increase in filters (8→16→32→64→128), which eliminates the early layer computational overhead. Moreover, the training time for the proposed LightMG-Net model is less because of the fewer trainable parameters of 2.6 million with fewer layers.

**Table 2:** The layered architecture of the proposed LightMG-Net model.

Layer name	Filter size	Kernels	Strides	Output size
Conv1	3 × 3	8	1	224 × 224 × 8
BNL	–	–	–	224 × 224 × 8
ReLU	–	–	–	224 × 224 × 8
MPL1	2 × 2	64	2	112 × 112 × 8
Conv2	1 × 1	16	1	112 × 112 × 16
BNL	–	–	–	112 × 112 × 16
ReLU	–	–	–	112 × 112 × 16
MPL2	2 × 2	32	2	56 × 56 × 16
Conv3	1 × 1	32	1	56 × 56 × 32
BNL	–	–	–	56 × 56 × 32
ReLU	–	–	–	56 × 56 × 32
MPL3	2 × 2	32	2	28 × 28 × 32
Conv4	1 × 1	64	1	28 × 28 × 64
BNL	–	–	–	28 × 28 × 64
ReLU	–	–	–	28 × 28 × 64
MPL4	2 × 2	32	2	14 × 14 × 64
Conv5	1 × 1	128	1	14 × 14 × 128
BNL	–	–	–	14 × 14 × 128
ReLU	–	–	–	14 × 14 × 128
MPL5	2 × 2	32	2	7 × 7 × 128
FCL1	–	–	–	512
Dropout	–	–	–	512
FCL2	–	–	–	4

We stuck with five, not four or six convolutional blocks. Incorporating six convolutional blocks, the model would start showing some extent of overfitting issues and require more computational resources. Furthermore, beyond a certain depth, the accuracy began decaying. If we used only four convolutional blocks, the model would be more computationally efficient, albeit at the cost of some degradation in performance indicators. Consequently, we employed only five convolutional blocks.

### Hyperparameter tuning of LightMG-Net using grey wolf optimization

The GWO has just been introduced by the Swarm Intelligence Algorithm^35^. The GWO algorithm is inspired by the social intelligence exhibited by grey wolf packs in their hunting strategies and leadership hierarchy. Grey wolves often inhabit groups consisting of roughly 5 to 12 individuals. A primary characteristic of this pack is social hierarchy. In pursuit of prey and upholding order within the pack, the Grey Wolf adheres to specific procedures categorized as social hierarchy, encirclement, harassment, and attack, which are further divided into four classifications: alpha, beta, omega, and delta. The purpose is to identify the minimum landscape. The alpha wolf makes crucial decisions for the pack, including hunting and choosing a residence, while the beta wolf conveys the alpha’s words to the other wolves. Delta is used to assist the alpha and beta wolves. Omega represents the final tier inside the pack, where wolves are allowed to consume food last. This is the paramount pack, as it may encounter internal issues in the absence of omega wolves. GWO uses this principle to rank the solutions and update their locations. The optimal mathematical model for the social hierarchy of wolves identifies alpha as the best-suited solution, followed by beta and delta as the secondary and tertiary solutions, respectively. The GWO algorithm’s optimization is guided by alpha, beta, and delta. These three wolves are being pursued by the omega wolves.

The grey wolf surrounds its prey throughout the hunt. Equations (12) and (13) are formulated to represent it mathematically:

12 $$\vec{D} = \left\| {\overrightarrow {C} \cdot \overrightarrow {{X_{P} \left( t \right)}} - \overrightarrow {X \left( t \right)} } \right\|$$

13 $$\overrightarrow {{X \left( {t + 1} \right)}} = \overrightarrow {{X_{P} \left( t \right)}} - \overrightarrow {A} \cdot \vec{D}$$

where A and C indicate the coefficient vectors, t denotes the current iteration, X represents the grey wolf position vector and X_P_ denotes the prey’s position vector. The mathematical equations of vectors A and C are depicted in equations (14) and (15), respectively

14 $$\vec{A} = 2\vec{a}.\overrightarrow {{r_{1} }} - \vec{a}$$

15 $$\vec{C} = 2\overrightarrow {{r_{2} }}$$

where a signifies a decreasing parameter and r_1_, and r_2_ represent the random parameters that lie in 0 and 1. As noted previously, the alpha, beta, and delta are the three best solutions that update first, and then the position of other search agents is updated as shown in Eqs. (16) and (17).

16 $$\overrightarrow {{D_{ \propto } }} = \left\| {\overrightarrow {{C_{1} }} \cdot \left( {\overrightarrow {{X_{ \propto } }} } \right) - \vec{X}} \right\|,\overrightarrow {{D_{\beta } }} = \left\| {\overrightarrow {{C_{2} }} \cdot \left( {\overrightarrow {{X_{\beta } }} } \right) - \vec{X}} \right\|, \overrightarrow {{D_{\delta } }} = \left\| {\overrightarrow {{C_{3} }} \cdot \left( {\overrightarrow {{X_{\delta } }} } \right) - \vec{X}} \right\|$$

17 $$\overrightarrow {{X_{1} }} = \left( {\overrightarrow {{X_{ \propto } }} } \right) - \overrightarrow {{A_{1} }} \cdot \left( {\overrightarrow {{D_{ \propto } }} } \right),\overrightarrow {{X_{2} }} = \left( {\overrightarrow {{X_{\beta } }} } \right) - \overrightarrow {{A_{2} }} \cdot \left( {\overrightarrow {{D_{\beta } }} } \right),\overrightarrow {{X_{3} }} = \left( {\overrightarrow {{X_{\delta } }} } \right) - \overrightarrow {{A_{3} }} \cdot \left( {\overrightarrow {{D_{\delta } }} } \right)$$

18 $$\overrightarrow {{X\left( {t + 1} \right)}} = \frac{{\vec{X}_{1} + \vec{X}_{2} + \vec{X}_{3} }}{3}$$

The grey wolf ends its hunt by attacking its prey when the prey stops their movement. The mathematical model indicates the approach to the prey by reducing the value of a. A variation is likewise diminished by ấ with A being randomly chosen from the interval [-2a, 2a], where the value decreases from 2 to 0 throughout the iterations. The GWO algorithm enables its search agents to adjust their positions according to the alpha, beta, and delta locations and attack the prey as shown in Eq. (18).

## Results

This section is divided into three subsections: a comparison of results with various optimization strategies, a class-wise result comparison, and the results for various lightweight models.

### Experimental environment

The proposed model was executed on MATLAB R2024b using DESKTOP-U4H7MEQ, Intel (R) Xeon (R) W-2133 GPU @ 3.60 GHz, with 64 GB RAM. This work employs 4475 preprocessed fundus images from each class for the multiclass diagnosis of RD disease. Of the total 4181 images employed for training, the remaining 294 images are used to test the proposed LightMG-Net model to get improved classification outcomes. The optimized hyperparameter configuration of the proposed model using GWO to improve the proposed LightMG-Net model’s convergence ability, as shown in Table 3.

**Table 3:** Optimized hyperparameters of the proposed LightMG-Net model.

Hyperparameters	Momentum	Learning rate	Epochs	L2Regularization
Values	0.949	0.0049	7	0.0029

### Performance indicators

This subsection adopted seven assessment indicators to assess the efficacy of the proposed LightMG-Net model. These performance indicators are AUC, sensitivity, specificity, accuracy, precision, F1-score, and Matthews correlation coefficient (MCC) and their corresponding mathematical formulas are shown in equations (19)-(24).

19 $$Sensitivity\left( {Recall} \right) = \frac{TP}{{TP + FN}}$$

20 $$Specificity = \frac{TN}{{TN + FP}}$$

21 $$Accuracy = \frac{TP + TN}{{TP + TN + FP + FN}}$$

22 $$Precision = \frac{TP}{{TP + FP}}$$

23 $$F1{\text{ - score}} = 2*\frac{Recall*Precision}{{Recall + Precision}}$$

24 $${\text{MCC}} = \frac{{\left( {TP \cdot TN} \right) - \left( {FP \cdot FN} \right)}}{{\sqrt {\left( {TP + FP} \right)\left( {TP + FN} \right)\left( {TN + FP} \right)\left( {TN + FN} \right)} }}$$

**Fig. 5:**
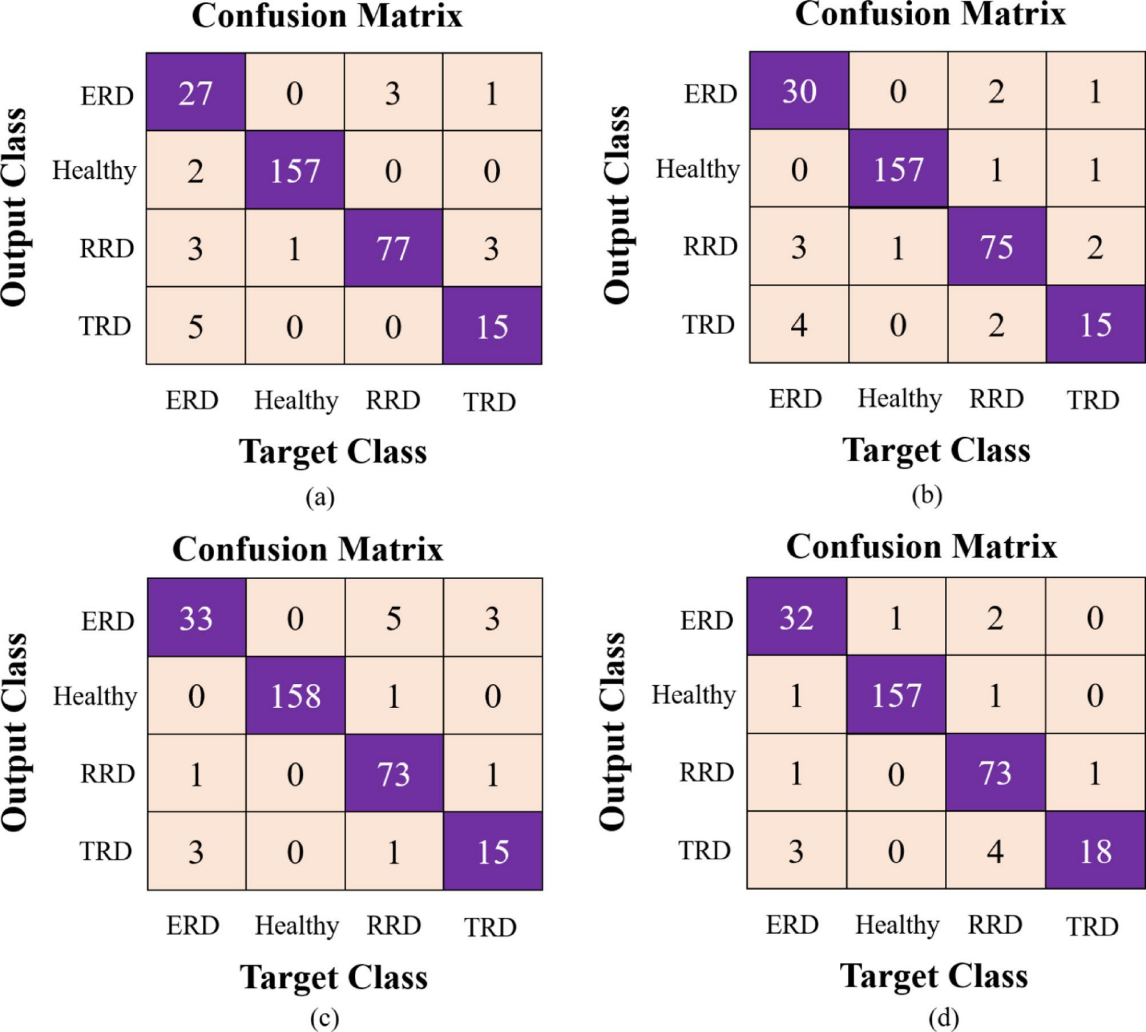
CMs of (**a**) GA, (**b**) PSO (**c**) WOA, and (**d**) the proposed LightMG-Net model.

**Fig. 6:**
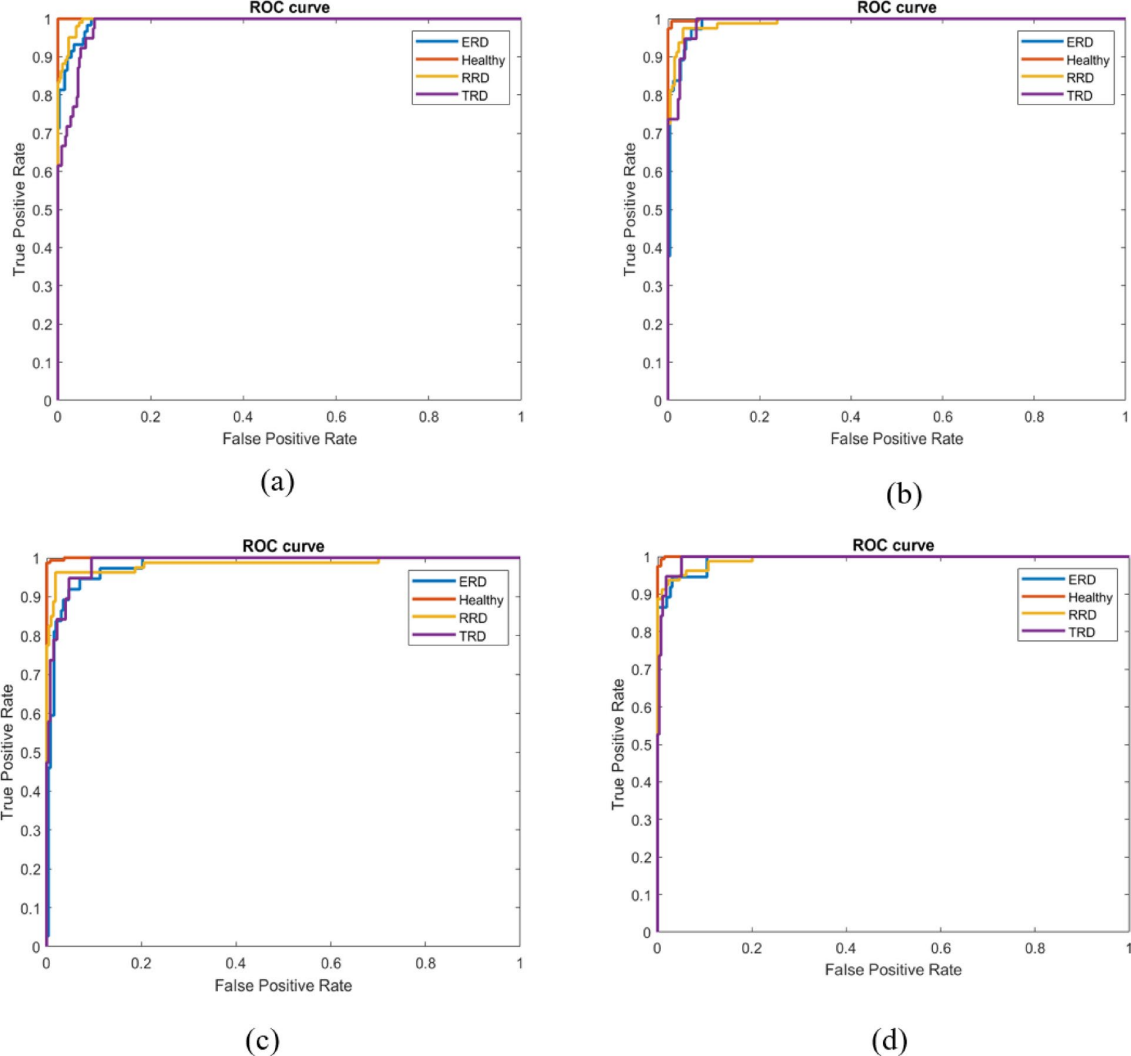
ROC curves of (**a**) GA, (**b**) PSO (**c**) WOA, and (**d**) the proposed LightMG-Net model.

**Fig. 7:**
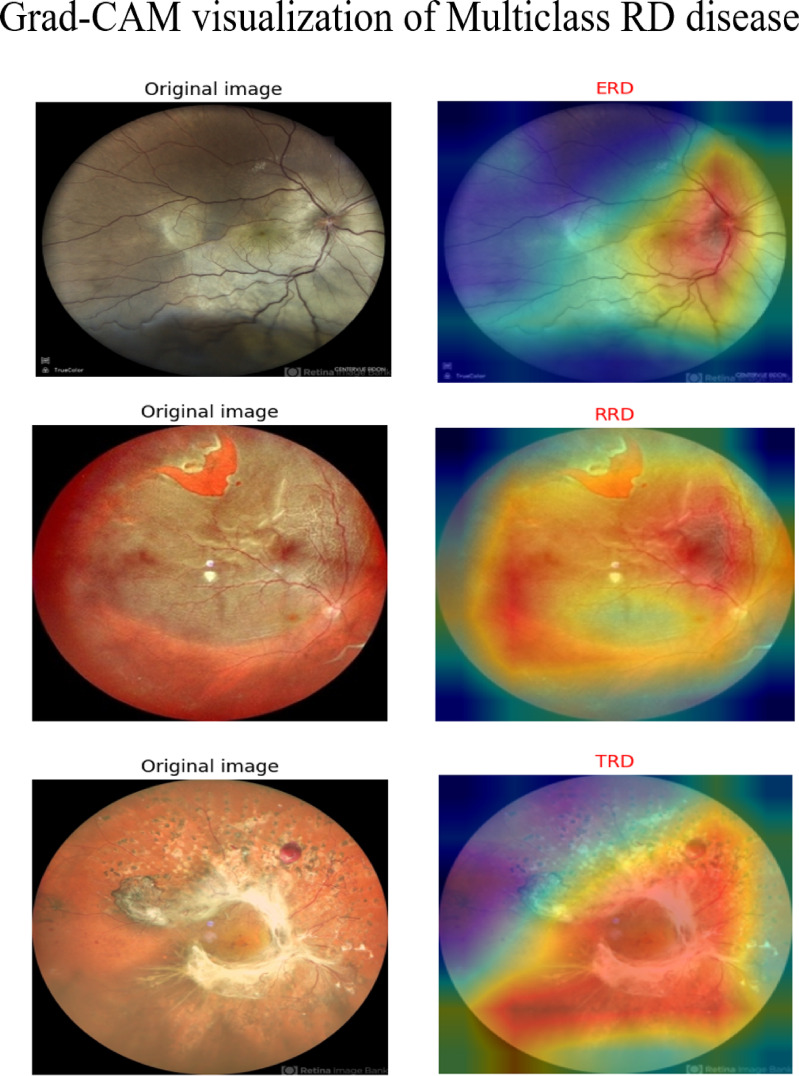
Grad-CAM visualization of multiclass grading of RD disease.

True Positive (TP), True Negative (TN), False Positive (FP), and False Negative (FN) constitute the four fundamental elements utilized in the calculation of the above indicators. Additionally, we showcase the outcomes of the proposed model using three representation graphs, the confusion matrix (CM), the ROC curve and Gradient-weighted Class Activation Mapping (Grad-CAM) visualization method as provided in Figs. 5d, 6d and 7. Grad-CAM elucidates the portions of the input image that most significantly impacted the model’s decision-making. Grad-CAM improves clinical interpretability by emphasizing disease-specific patterns, such as retinal lesions such as retinal lesions, fluid accumulation, and unwanted tissue growth. The orange hue in Fig. 7 denotes the disease-discriminative areas that facilitate model predictions, strengthen clinical validation, and aid in error analysis. Furthermore, it verifies that the model is not basing decisions on artifacts or noise.

### Comparative analysis

This subsection offers a comparative analysis of the proposed methodology for multiclass categorization of RD fundus images. Comparisons are conducted with various optimization algorithms, class-wise result analysis, lightweight model evaluations, K-fold cross-validation, and ablation examination based on the previously described performance indicators.

#### Comparison of the model’s performance using different optimizers

To thoroughly compare the performance of the proposed multiclass lightweight LightMG-Net model, which utilized three meta heuristic optimization algorithms, such as the genetic algorithm (GA)^36^, Particle swarm optimization (PSO)^37^ and whale optimization algorithm (WOA)^38^, under identical training conditions. The accuracy metrics for each optimization algorithm were recorded and analyzed to compare their effectiveness in the multiclass classification of RD disease. Moreover, the results are also compared with two visual representation graphs, such as the CM and ROC curve, as given in Figs. 5 and 6, respectively. The resulting accuracies are as follows: GA: 94.10%, PSO: 94.22%, WOA: 94.90% and GWO: 95.24%, as shown in Table 4. The performances of the GA, PSO and WOA are approximately similar in terms of classification accuracy in the range of 94%. GWO surpasses GA, PSO, and WOA in accuracy owing to its leader-centric social hierarchy, adaptive exploration-exploitation balance, varied search methodologies, simplicity of fewer parameters, and multi-leader strategy that mitigates premature convergence. These variables effectively enable GWO to identify optimal solutions with improved classification accuracy.

**Table 4:** Comparison of proposed LightMG-Net model performance with various optimization algorithms.

Metric	GA	PSO	WOA	GWO
Accuracy (%)	94.10	94.22	94.90	95.24
Sensitivity (%)	93.95	94.10	94.36	95.10
Specificity (%)	98.00	98.20	98.60	98.90
Precision (%)	93.81	94.35	95.10	95.75
F1-Score (%)	93.70	94.21	95.00	95.31
AUC	0.9876	0.9938	0.9914	0.9947

#### Class-wise result comparison of the proposed lightweight LightMG-Net

Table 5 shows the class-wise performance comparison of the proposed LightMG-Net model regarding AUC, sensitivity, specificity, accuracy, precision, F1 score and MCC. Based on the statistical data, the proposed LightMG-Net model obtained the highest accuracy of 99.51% for the Healthy class, which is the most significant subset of the dataset. This class did exceptionally well on all the metrics, with a precision of 98.75% and a perfect recall score of 99.41%, giving it an impressive F1 score of 99.10%, Specificity of 98.50% and AUC of 0.9998, further confirmed the robust ability of the model to identify healthy retinal images correctly. When talking about the RRD class, it is shown that it performed exceptionally well across each performance metric, whether it is accuracy, sensitivity, specificity, precision, F1-score, AUC and MCC. The ERD class achieved an accuracy of 87.80%, specificity of 86.50%, and F1-score of 88.90%, marginally inferior to healthy and RRD classes. Additionally, it achieved improved specificity and AUC scores. In addition, the TRD class achieved better accuracy, sensitivity, specificity, and AUC but marginally worse precision, F1-score and MCC values than other classes. The diminished performance may be attributed to the misclassification of most ERD classes as TRD classes and vice versa, owing to the resemblance in the structure of RD lesions.

**Table 5:** Class-wise performance metrics of the proposed LightMG-Net model.

Class	Accuracy (%)	Sensitivity (%)	Specificity (%)	Precision (%)	F1-score (%)	AUC	MCC
ERD	87.80	86.50	98.80	91.40	88.90	0.9922	0.8737
Healthy	99.51	99.40	98.50	98.70	99.10	0.9998	0.9790
RRD	92.50	91.20	99.10	97.30	94.20	0.9927	0.9300
TRD	94.50	94.70	97.50	72.00	81.80	0.9943	0.8130

#### Performance comparison using stratified K-fold cross-validation

This work utilizes the cross-validation (CV) approach that employs distinct data subsets to assess the model’s performance. It involves partitioning datasets into training and testing portions across several iterations. CV is especially beneficial for imbalanced datasets, as it prevents the model’s training or testing on folds that are predominantly biased toward a single class. In this work, we adopt a K value of 10. As a result, the RD fundus images are categorized into ten subsets for each class. In each iteration of CV, k-1 subsets are used for training, while another subset is used for model testing. Table 6, showcases the performance metrics of the proposed model, including accuracy, sensitivity, specificity, precision, F1-score, AUC, and error rate using k-fold CV repeatedly computed for K scenarios.

**Table 6:** Result of K-fold cross-validation.

Testing fold	Accuracy (%)	Sensitivity (%)	Specificity (%)	Precision (%)	F1-score (%)	AUC	Error rate
Fold1	90.90	84.20	97.31	82.87	83.53	0.9740	0.0909
Fold2	93.93	86.16	98.18	86.90	86.53	0.9922	0.0606
Fold3	90.90	83.17	97.12	85.53	84.34	0.9918	0.0909
Fold4	96.97	92.58	98.95	97.50	94.97	0.9921	0.0303
Fold5	90.81	83.08	96.83	86.17	84.60	0.9880	0.0918
Fold6	94.89	89.81	98.27	89.20	89.50	0.9916	0.0510
Fold7	94.89	86.57	97.62	96.87	91.43	0.9951	0.0510
Fold8	94.89	87.73	98.46	87.73	87.73	0.9946	0.0510
Fold9	93.87	85.64	98.20	85.64	85.64	0.9881	0.0612
Fold10	89.79	77.70	96.67	81.95	79.77	0.9836	0.1020
Overall	93.19	85.66	97.76	88.04	86.80	0.9891	0.0680

#### Performance comparison with various lightweight LightMG-Net models

A performance comparison of the proposed lightweight LightMG-Net against four prevalent pre-trained deep neural networks such as EfficientNetb0, MobileNetV1, ShuffleNetV2, and MobileNetV2, is presented in Table 7 to assess the effectiveness of the suggested multiclass lightweight CNN model. The EfficientNetb0 model had the least performance and demonstrated inferior classifications relative to the other models. MobileNetV1 exhibited superior performance relative to EfficientNetb0, althoughinferiortoShuffleNetandMobileNetV2. Upon assessment, ShuffleNetV2 and MobileNetV2 exhibit nearly identical performance characteristics. The proposed multiclass lightweight CNN model surpasses all other networks, enhancing performance across all four classes and metrics. The reason behind that is our proposed model integrates image and feature-specific handcrafted features (e.g., AM-FM decomposition, Gabor filtering, and HSV-based texture analysis), to capture rich and clinically meaningful image characteristics that typical pre-trained CNNs may overlook. Moreover, the Grey Wolf Optimization additionally improves feature selection, eliminating redundant features and enhancing decision boundaries. That’s the reason behind the better accuracy of the proposed model. As far as speed and model size are concerned, the proposed lightweight model utilized fewer layers with fewer trainable parameters of 2.6 million. Due to this reason, the training speed of the proposed model is faster, along with a smaller model size than other prior lightweight models.

**Table 7:** Performance comparison with various lightweight models.

Structure	Accuracy (%)	Specificity (%)	Sensitivity (%)	Precision (%)	F1-score (%)	AUC	Parameters (millions)	Computational time (mins)
EfficientNetb0	90.80	90.81	97.70	91.20	90.23	0.9825	5	22:12
MobileNetV1	91.81	91.56	96.90	91.73	91.70	0.9895	4.2	15:43
ShuffleNetV2	92.51	91.26	97.50	91.49	91.23	0.9878	5.4	25:25
MobileNetV2	93.50	93.35	98.00	93.40	93.50	0.9899	3.4	13:48
LightMG-Net	95.24	95.10	98.90	95.73	95.31	0.9947	2.6	9:00

#### Experimental results based on Datasets to showcase the generalizability of the proposed model

This study compares the performance of online and offline databases, as illustrated in Table 8. Offline data samples were obtained from Silchar Medical College and Hospital in Silchar, India. A total of 100 distinct types of RD fundus images have been acquired from this hospital to develop the proposed model. Of the 100 images, 25 fundus images are from healthy patients, while the remaining 75 are categorized into RRD, ERD, and TRD, with 25 images allocated to each for the implementation of the suggested multiclass RD diagnostic model. The online database comprises 990 fundus images. Of the 990 images, 600 are from healthy individuals, while the remaining 390 consist of 98 from ERD, 207 from RRD, and 65 from TRD, as illustrated in Table 1. Table 8 indicates that online databases exhibit superior performance compared to offline databases, attributed to the reduced sample size of the offline database. However, it can be stated that despite the absence of an offline database, we attained satisfactory performance metrics due to enhanced clarity of RD lesions, good contrast, improved lighting effects, and reduced artifacts.

**Table 8:** Performance comparison with online and offline data samples.

Database	Accuracy (%)	Specificity (%)	Sensitivity (%)	Precision (%)	F1-score (%)	AUC
Online (LightMG-Net)	95.24	95.10	98.90	95.73	95.31	0.9947
Offline (LightMG-Net)	86.28	82.93	89.46	82.67	81.26	0.9656

#### Annotation process of retinal detachment

The annotation of retinal detachment (RD) in fundus images was conducted under the guidance of experienced senior ophthalmologists to guarantee clinical precision and dependability. Each image was meticulously analyzed, and areas exhibiting indications of RD were outlined with specialist annotation tools. According to the study requirements, the annotation was conducted at both the picture level (indicating the presence or absence of RD) and the region level (detailed boundary segmentation of detached areas). To reduce subjectivity, multiple ophthalmologists independently annotated the images, and any conflicts were reconciled through consensus or adjudication by a senior expert. The final annotations were preserved as binary masks and structured files, facilitating their immediate application in the training and validating deep learning models. Inter-rater reliability was evaluated by statistical metrics, including Cohen’s Kappa, to guarantee the generalizability, robustness, and consistency of the labeled dataset.

**Fig. 8:**
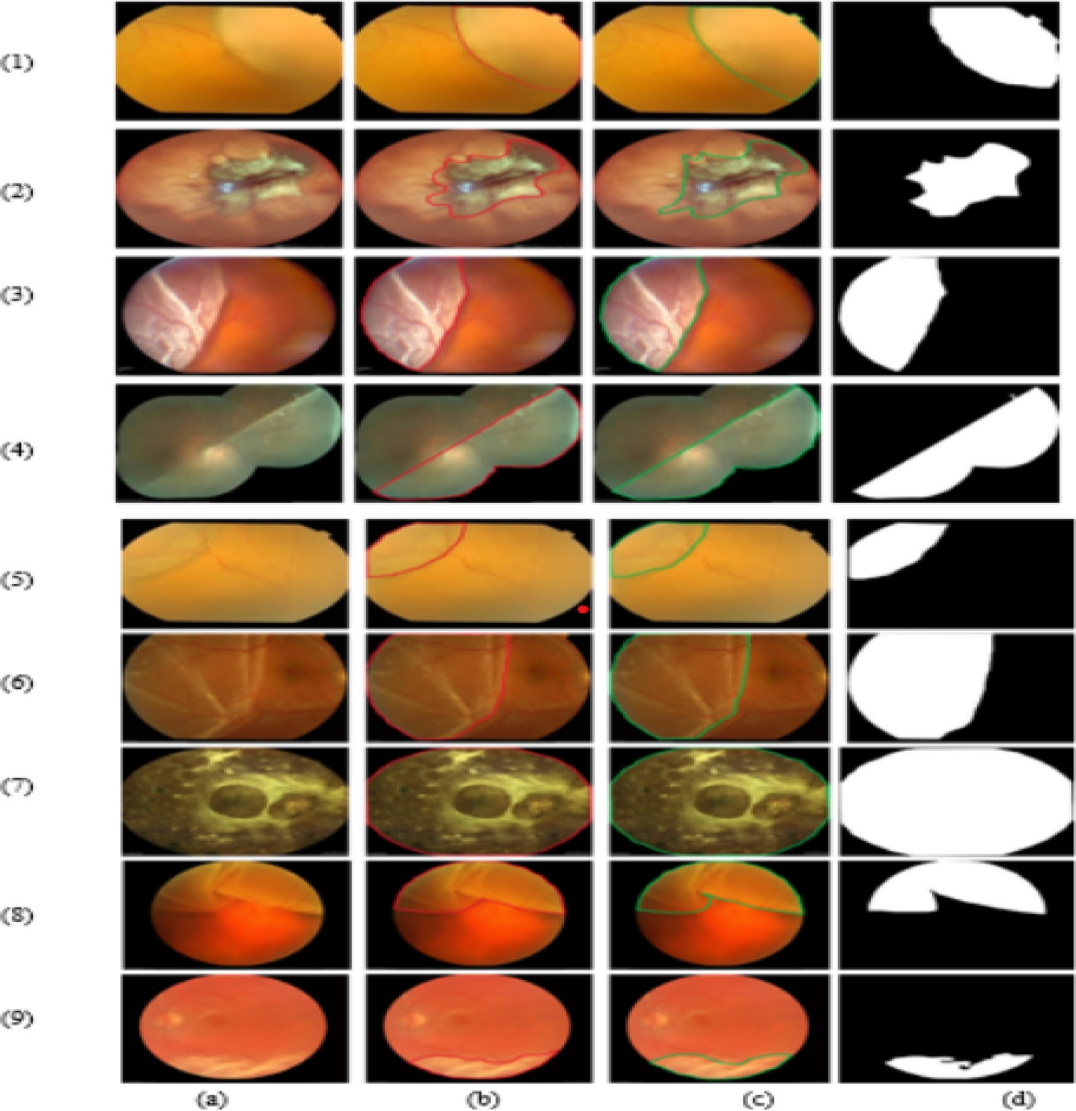
Annotation process of RD fundus image (**a**) Original image, (**b**) Ground truth image, (**c**) RD lesions fundus image annotation by experts and (**d**) results obtained by proposed model.

In this study, we utilized region level annotation procedure to ensure that the ground truth labels precisely depict diseased locations with specialist annotation tools named VGG Image Annotator as shown in Fig. 8. In addition to that the inter-rater reliability of this work is evaluated by performance indicator named Cohen’s Kappa as shown in Table 9.

**Table 9:** Annotation procedure of RD fundus images.

Image set	Cohen’s Kappa	Interpretation of Cohen’s Kappa value
1–3	0.81	Almost perfect agreement by two annotator
4–6	0.78	Substantial agreement
7–9	0.86	Almost perfect agreement by two annotator

Cohen’s Kappa (κ) is a statistical metric for assessing inter-rater reliability (agreement) in categorical data. It is extensively utilized in medical image annotation, such as RD fundus images labelling, to assess the degree of agreement between two experts beyond mere chance. The mathematical formula for Cohen’s Kappa is given in Eq. (25).

25 $$\upkappa = \frac{{p_{o} - p_{e} }}{{1 - p_{e} }}$$

where $p_{o}$ represents observed agreement (the ratio of instances in which both evaluators concur) and $p_{e}$ represents expected agreement by random occurrence.

The annotator classes for this work categories into four categories:

1. Class 0: ERD
2. Class 1: Healthy
3. Class 2: RRD
4. Class 3: TRD

#### Ablation study

This study conducted ablation experiments to verify that the selection of an optimized LightMG-Net and image and feature-oriented handcrafted techniques have positively influenced the classification outcomes. The ablation study was conducted on six specific scenarios, and the outcomes of the ablation experiment are presented in Table 10.

1. Light-weight CNN
2. Light-weight CNN + handcrafted techniques
3. Light-weight CNN + GWO
4. Image-oriented handcrafted (IOH) + Light-weight CNN + GWO
5. Feature-oriented handcrafted (FOH) + Light-weight CNN + GWO
6. LightMG-Net model

**Table 10:** Ablation experiment based on five different scenario.

Structure	Accuracy (%)	Specificity (%)	Sensitivity (%)	Precision (%)	F1-score (%)	AUC
CNN	87.69	86.50	95.59	86.71	86.39	0.9665
CNN + handcrafted	89.63	89.21	94.95	87.69	87.45	0.9711
CNN + GWO	90.20	90.31	97.90	90.80	90.40	0.9830
IOH + CNN + GWO	92.44	92.39	98.70	92.90	92.69	0.9934
FOH + CNN + GWO	93.99	93.91	98.40	93.86	93.80	0.9883
LightMG-Net model	95.24	95.10	98.90	95.73	95.31	0.9947

According to Table 10, it can be demonstrated that the CNN optimized with GWO shows a certain degree of improvement compared with the individual CNN model and the handcrafted techniques, including all performance metrics such as accuracy, sensitivity, specificity, precision, F1-score, and AUC. This is because the GWO optimization algorithms can more effectively achieve optimal solutions with fewer parameters and multi leader strategies that prevent premature convergence. Utilizing the image-oriented handcrafted techniques results in marginal improvements across all six metrics. This occurs because image-centric handcrafted methods concentrate on the pertinent region of interest, specifically diseased retinal detachment lesions, while excluding extraneous features. Moreover, incorporating feature-level handcrafted techniques facilitates the extraction of rich features, texture, and intensity of the infected retinal disease lesions, resulting in superior performance compared to previous component combinations and improving classification accuracy for multiclass grading of RD disease. This observation indicates that each module is advantageous for improving model performance, particularly the third and fourth modules, which significantly contribute to the improved effectiveness of the proposed LightMG-Net model.

## Discussion

This work presents a lightweight CNN model optimized with GWO for better feature extraction and multiclass classification of RD fundus images. This work only considers the informative portion of the training images, which yielded better performance matrices than other RD methodologies. The assessment indices state that the proposed LightMG-Net architecture is a promising tool for detecting the severity of RD disease with notably higher accuracy than other RD models. Furthermore, the proposed work also showcases better discrimination ability to accurately classify each form of RD fundus images with less misclassification.

Some specific advantages of handcrafted features over other deep learning models are listed below:

- Handcrafted features (both image-oriented and feature-oriented) provide explicit visual interpretability, clearly correlating with clinical patterns such as lesions or fluid accumulation, hence enhancing physician trust and disease clarity. That is something missing in deep learning models due to their black-box nature.
- Deep learning methods necessitate extensive, labeled datasets for effective generalization. Conversely, handcrafted features can attain satisfactory performance with limited datasets.
- Handcrafted systems, characterized by minimal parameters and direct control over feature selection, exhibit a reduced susceptibility to overfitting, especially in datasets with an inadequate number of samples or significant class imbalance. In contrast, deep learning models possess millions of parameters, rendering them costly and potentially resulting in significant overfitting problems when data samples are inadequate.

A significant advantage of the proposed model is that it uses image and feature-oriented handcrafted techniques that yield the most informative retinographic samples for feature extraction. These techniques enable our model to localize the ROI to correctly learn minute and unnoticeable retinal image features for multiclass RD grading. Moreover, hyperparameter tuning using GWO yields optimized hyperparameter values and enhances the model’s predictive accuracy.

Additionally, a notable advantage of the proposed model is its ability to maintain a low computing burden during training while automatically extracting the discriminative features from the training datasets. This yields a fast and reliable approach to training the model. Besides this, some disadvantages have also been noticed. One of the disadvantages is that GWO has many controlling parameters that must be selected carefully to solve the problem of a vast number of parameters and features. Additionally, this work utilizes a limited dataset to implement the proposed methodology. Utilizing a small dataset may restrict the model’s generalization ability across varied populations. Consequently, the model’s robustness and generalizability must be verified with larger, more diversified datasets that accurately reflect real-world scenarios.

## Conclusion

The early identification of RD is challenging due to the relatively small RD lesion size and varying structure. Identifying RD disease is essential for determining the cause of visual impairment and total vision loss in the human eye. This work proposes a LightMG-Net model based on CNN for multiclass grading RD disease. The proposed model utilized image-level and feature-level handcrafted techniques for better feature analysis, followed by hyperparameters tuning the LightMG-Net model using the GWO approach to get optimal solutions for the severity grading of RD disease. This study utilized four widely recognized RD disease datasets, Retinal Image Bank, Cataract Image Dataset, Kaggle, and Eye Diseases Retinal Images, to validate the performance of this work. The experimental findings indicated a classification accuracy of 95.24%, sensitivity of 95.10%, and specificity of 98.90%, which outperformed other prior benchmark models. In the future, we plan to utilize the hybrid optimization techniques (GWO with other optimization algorithms) to address the problem of controlling the numerous parameters in GWO. We also aim to evaluate the performance of the proposed LightMG-Net model using extensive databases, both in online and real-time scenarios. Additionally, we intend to deploy graph neural network models to facilitate the RD multiclass grading, utilizing a minimal number of trainable parameters and reduced computational demands.

## Data Availability

The datasets used and/or analyzed during the current study available from the corresponding author on reasonable request.
